# Cure of Itch in Half an Hour

**Published:** 1856-10

**Authors:** 


*Cure of Itch in Half an Hour.*

Dr. E. Smith, at a meeting of the London Medical Society, called attention to an article in the *Gazette Hebdomadaire*, by Dr. Bourguignon, in which is a confirmation of the value of the treatment of itch, in Belgium, by sulphur, combined with lime, in a liquid form. The remedy is prepared by boiling one part of quick lime with two parts of sublimed sulphur, in ten parts of water, until the two former are perfectly united. During the boiling it must be constantly stirred with a piece of wood, and, when the sulphur and lime have combined, the fluid is to be decanted and kept in a well-stoppered bottle. A pint of the liquid is sufficient for the cure of several cases. It is sufficient to wash the body well with warm water, and then to rub the liquid into the skin for half an hour. As the fluid evaporates, a layer of sulphur is left upon the skin. During the half hour the acarus is killed, and the patient is cured. It is only needful then to wash the body well, and to use clean clothes. In Belgium, the treatment is introduced by first rubbing the body for half an hour with black soap; but this does not appear to be necessary. The only essential act is that of the careful application of the fluid sulphur. The lime is of no importance in the treatment, except to render the sulphur soluble, and such would probably be the case if potass or soda were employed. The chief point in the plan thus employed, which is an improvement upon the mode of application of sulphur in substance with lard, is the more ready absorption of the remedy, and consequently the more certain and quick destruction of the insect, by using sulphur in a fluid form. In so disgusting a disease it must be of great moment to be able to cure it in half an hour.—*Assoc. Med. Jour.*

